# Technical challenges to surgical clipping of aneurysmal regrowth with coil herniation following endovascular treatment – a case report

**DOI:** 10.1186/1752-1947-1-168

**Published:** 2007-12-04

**Authors:** Promod Pillai, Aftab Karim, Anil Nanda

**Affiliations:** 1 Department of Neurosurgery, Louisiana State University Health Sciences Center, Shreveport, Louisiana 71130, USA

## Abstract

In recent years, technical developments have made endovascular procedures attractive therapeutic options and enabled the endovascular surgeon to redefine the management of cerebral aneurysms. However, as the number of aneurysms undergoing endovascular therapy has grown, so has the number of patients with incompletely treated aneurysms who are presenting for further management. In cases of failure of endovascular treatment caused by either incomplete occlusion or regrowth of the aneurysm, a complementary treatment is often necessary. Surgical treatment of these patients is challenging. We present a case of a ruptured posterior cerebral artery aneurysm treated initially with endovascular coiling that left behind significant residual aneurysmal sac. Regrowth of the aneurysm documented on follow-up was treated surgically. At surgery, the coil was found to have herniated through the aneurysmal sac into the subarachnoid space, and the aneurysm was successfully clipped without removing the coils. We review the regrowth of aneurysms following endovascular therapy and potential problems and challenges of surgically managing these lesions.

## Introduction

In the last 2 decades, since the introduction of Guglielmi detachable coils (GDC), endovascular management of intracranial aneurysms has progressed significantly[1]. This treatment represents an interesting alternative for intracranial aneurysms with difficult surgical access. However, as a result of the growing number of intracerebral aneurysms treated with endovascular coils, a new group of patients has arisen who have undergone endovascular treatment that has resulted in incomplete occlusion or aneurysmal regrowth that often requires complementary treatment [2-11]. Surgical treatment of aneurysms following failed endovascular treatment is not simple and is often hazardous. We present a case of aneurysm following failed endovascular treatment that required further surgery.

## Case presentation

**Figure 1:**
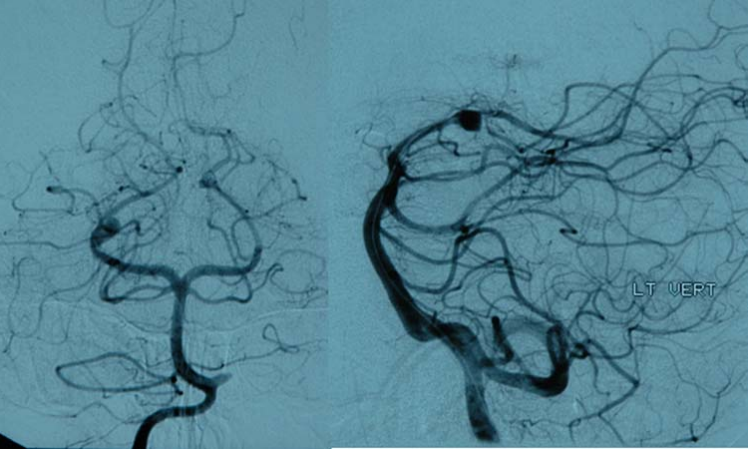
Arteriogram showing aneurysm of the posterior cerebral artery.

**Figure 2:**
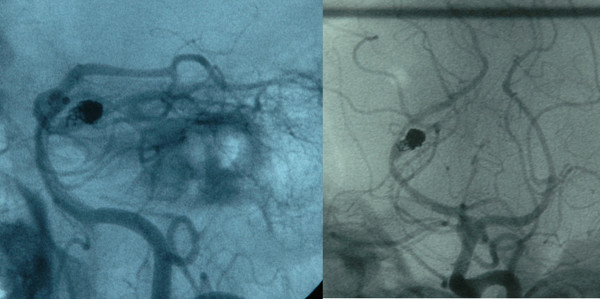
Post-embolization arteriogram showing coiled aneurysm of the posteriorcerebral artery with residual aneurysmal sac.

**Figure 3:**
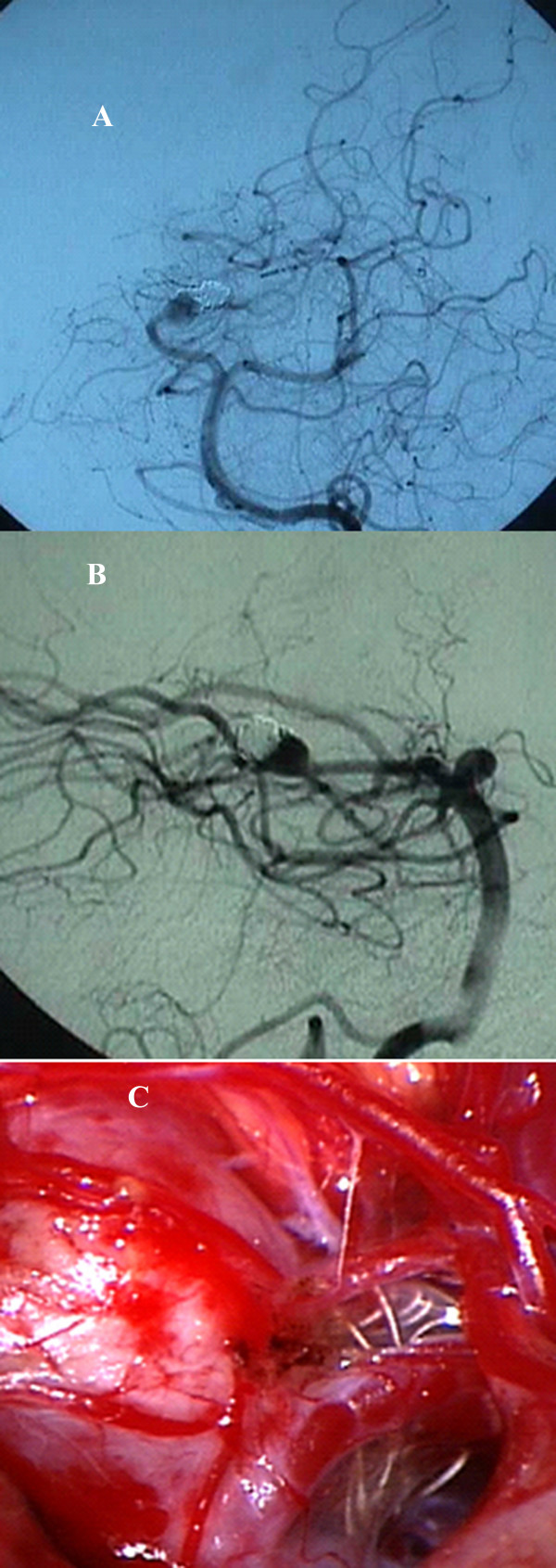
A, B. Follow-up arteriogram shows regrowth of the aneurysmal sac. C. Operative picture showing the aneurysm with coil herniation into the subarachnoid space.

A 34-year-old woman was initially treated for a subarachnoid hemorrhage at another center. Imaging by cerebral arteriography revealed a saccular aneurysm arising at the second segment of the right posterior cerebral artery (Fig. 1), and she underwent endovascular treatment of the aneurysm with GDCs. Post-embolization arteriogram revealed coiling of the aneurysm with a residual aneurysm sac (Fig. 2). Despite the significant residual aneurysmal sac, the primary treating physician decided to follow her angiographically. Angiographic follow-up 18 months later revealed enlargement of the residual aneurysm (Fig. 3A, B), and she declined a new endovascular procedure and opted to come to our facility for further management.

At our center, the patient underwent a right subtemporal surgical approach and microsurgical clipping of the aneurysm. During surgery, no attempt was made to remove endovascular coils that had herniated through the aneurysmal sac into the subarachnoid space (Fig. 3C). The relatively immobile coil mass made it difficult to define the aneurysm sac. The parent artery was temporarily occluded to define the aneurysm complex. The patient tolerated the procedure well and recovered completely with no new neurological deficits.

## Discussion

Treatment options for intracranial aneurysm are no longer limited to craniotomy with aneurysm clip occlusion. The rapidly evolving technical advances in endovascular neurosurgery have popularized this technique for aneurysm treatment. Careful patient selection is paramount for successful outcome with endovascular treatment. Generally, endovascular options are considered when the aneurysm involves the posterior circulation and anatomic considerations, medical conditions, or poor neurological grade do not recommend surgical intervention. However, with the growing use of endovascular therapy, neurosurgeons encounter more patients with incompletely treated intracranial aneurysms. Poor patient selection, incomplete treatment, and aneurysm regrowth contribute significantly to the failure of endovascular treatment [2-11]. A rate of 24.8 to 44% partial occlusion after coiling is reported [4]. The International Subarachnoid Aneurysm Trial (ISAT) was a randomized study comparing neurosurgical clipping and endovascular coiling in patients with ruptured intracranial aneurysms. After coiling, 66% of follow-up angiograms showed complete angiographic occlusion, 26% showed subtotal occlusion or a neck remnant, and 8% showed incomplete occlusion with aneurysm refilling [8]. In our case, initial treatment failed as a result of incomplete occlusion, and an unstable residual aneurysm grew over time. Though clinical relevance of recanalization in partially coiled aneurysms remains unclear, it is essential to follow these patients carefully.

## Surgical treatment of previously coiled aneurysms

Failure of initial endovascular approach to an aneurysm requires reassessment of clinical data and redesign of treatment strategy. Important considerations include the patient's neurological status and general medical condition, the location and anatomical structure of the aneurysm, the degree of neck occlusion, and the time elapsed since the last endovascular procedure [9,10].

Surgery is preferred if vascular anatomy or aneurysm configuration is unfavorable for further endovascular embolization, but operation of these aneurysms is not always simple. Protrusion of the coils inside the aneurysm neck or even inside the parent artery could make clipping very hazardous and increase the risk of stenosis of the parent artery, especially when the aneurysm is associated with atheromatous plaque on the parent vessel [8]. These factors increase the operative morbidity and mortality of the lesions.

An alternative to clipping the aneurysm, in some cases, may include wrapping it and ligating and coagulating its neck [4]. When clipping the aneurysm neck is difficult, some have proposed removing the protruding coils [3,6,8-10] or following the aneurysm angiographically until the neck is coil free and clipping can be conducted freely [4,5]. If coil removal is attempted, it is important to obtain proximal and distal control of the arteries to and from the aneurysm and to avoid pulling too vigorously and tearing the aneurysmal sac from the parent artery [5,8-10]. Sometimes it will be highly dangerous to attempt removing the coils, especially if coils have been in the aneurysm for more than a few weeks and developed a strong adhesion to the intra-aneurysmal thrombosis [8].

Gurian's group reported 8 aneurysms that required surgical procedures following endovascular treatment [5]; five were clipped; two, bypassed and trapped; and one managed with Hunterian ligation, all with good to excellent results. They observed that primary clipping is easier when aneurysms are free of coils at the neck; clipping of those with a coil mass in the neck is difficult and may require coil extraction. Thornton and associates [8] reported 11 patients who underwent surgery following endovascular coiling of an aneurysm with GDCs. They recommend that when coils have been in the aneurysm for a long time, they should be left in place and the neck be allowed to grow before clipping is attempted. Tirakotai and colleagues [9] reported a recent series of 8 patients who underwent surgical intervention for various indications following endovascular treatment. Complete obliteration was achieved in all cases, with excellent outcome in 6 cases. In cases of large, partially coiled aneurysms, in which visualization of the lesion is obstructed by a relatively immobile coil mass, intraoperative angiography is valuable to confirm clip position and assess patency of parent vessels.

In our case, interestingly, part of the coil mass was herniating into the subarachnoid space through a rent in the aneurysmal sac, and there were dense adhesions to the arachnoid as well as to the aneurysm. This made mobilization of the aneurysmal sac difficult. We temporarily occluded the parent artery before attempting to define the sac, and we made no attempt to remove the coils. Other authors have also reported extrafundal coils [3,5,6], which Horowitz's group attributes to fundal erosion by the coil mass or perforation during surgery [6]. In our case, we found a significant portion of coil mass to be herniating into the subarachnoid space. We propose that this phenomenon of fundal erosion by the coil mass and subsequent herniation of coil/s outside the aneurysmal sac results from the "water-hammer effect" of pulsatile blood flow into the remnant aneurysm. If the aneurysmal sac is partially occluded with coils, the residual sac is still in communication with general circulation, which subjects the coils to constant arterial pulsations. Over time, these transmitted pulsations can lead to enlargement of the aneurysmal sac with subsequent migration of the coils [7].

## Conclusion

The growing use of endovascular therapies has led to more patients with failed treatments presenting for further management. Though the clinical relevance of recanalization in partially coiled aneurysms remains unclear, it is essential that residual aneurysms be monitored long enough to infer information about the treatment's long-term outcome. This situation highlights the need for developing new guidelines for managing cerebral aneurysms and the absolute necessity for collaboration between endovascular and neurosurgical specialists to provide the best treatment for cerebral aneurysms.

## Abbreviations

GDC: Guglielmi detachable coils.

## Competing interests

The author(s) declare that they have no competing interests.

## Authors' contributions

PP and AK contributed to the paper's conception, acquisition and interpretation of data, and drafting and revision of the manuscript. AN was involved in revising the manuscript critically and gave final approval of the manuscript.

## Consent

Written consent was obtained from the patient for publication of the study.

## Appendix

### A. Patient details

- Age, 34 yrs

- Sex, female

- Country of residence, USA

### B. Clinical Details

- reason for presentation: subarachnoid hemorrhage

- primary diagnosis: subarachnoid hemorrhage

- secondary diagnosis: intracranial aneurysm

### C. Investigations & Pharmaceutical preparations

4-vessel cerebral angiography
